# Magnetic composite of γ-Fe_2_O_3_ hollow sphere and palladium doped nitrogen-rich mesoporous carbon as a recoverable catalyst for C–C coupling reactions

**DOI:** 10.1038/s41598-021-99679-x

**Published:** 2021-11-17

**Authors:** Masoume Malmir, Majid M. Heravi, Zahra Amiri, Kosar Kafshdarzadeh

**Affiliations:** Department of Chemistry, School of Physics and Chemistry, Alzahra University, PO Box 1993891176, Vanak, Tehran, Iran

**Keywords:** Catalyst synthesis, Heterogeneous catalysis

## Abstract

In this article, palladated-magnetic nitrogen doped porous carbon was prepared from nano magnetic **γ-**Fe_2_O_3_ hollow sphere (h-Fe_2_O_3_) with high specific surface area and pore volume. To the purpose, initially h-Fe_2_O_3_ was prepared and covered with glucose via hydrothermal treatment with subsequent polymerization of organic shell. The polymerization of melamine-resorcinol–formaldehyde (MRF) was achieved in the presence of Cl-functionalized glucose coated h-Fe_2_O_3_ (h-Fe_2_O_3_@glu-MRF). Next, the prepared magnetic core–shell hollow sphere was palladated followed by carbonization to yield Pd@h-Fe_2_O_3_@C introducing more pores in its structure. The resulted compound, Pd@h-Fe_2_O_3_@C, was fully characterized, showing that carbonization process expressively increased the specific surface area. The resulted Pd@h-Fe_2_O_3_@C was successfully used for promoting C–C coupling reactions under mild reaction conditions as a heterogeneous catalyst and its activity was compared with some prepared control catalysts. This novel catalyst was magnetically separated simply by a magnet bar and recycled and reused at least in five consecutive runs, without considerable loss of its activity. It is note mentioning that, high recyclability with low Pd leaching are another gains of this protocol.

## Introduction

Nowadays, metal-catalyzed cross-coupling reactions are considered and well-established strategy in synthetic organic chemistry due to their remarkable applications in the preparation of various intermediates for the mass production of agrochemicals, pharmaceuticals, and natural products^1,2^ as well as oligomers^3^ and conducting polymers^4,5^. The Pd-catalyzed cross-coupling, known as Stille, Buchwald, Sonogashira, Heck and Suzuki cross coupling reactions, has been documented as one of the most influential tools for the carbon–carbon bond formation. Palladium, especially in homogeneous catalysis is an unavoidable catalyst choice for such coupling reactions due several advantages such as selectivity, high synthetic yielding, and short reaction time^6,7^. Nevertheless, the recovery of homogeneous Pd complexes for the sake of reusing them from the reaction mixture is often problematic and usually could not be reused for the second run of reaction. Whereas, separation and recovery of catalysts, when expensive noble metal catalysts are being used or the case is in environmental concerns is definitely highly desired. Henceforth, heterogenization of catalysts are actually the striking solution to these glitches. In this regard a wide range of inorganic and organic substances have already been designed and employed for supporting dissimilar metal catalysts to create heterogeneous catalysts. Among these solid supports, nanomagnetic and mesoporous materials have attracted attention because to their exceptional properties such as relaxed recovery and high surface area^8–10^. In this regard, to enhance the specific surface area and decrease the content of active components, building a novel heterogeneous catalyst on a suitable metal support has been proposed. The hollow magnetic nanoparticles with exceptional merits for example low density, high specific surface area, great pore volume, and mechanical stability is an appropriate substrate for cumulative the selectivity and successful catalyst stability^11^.

Alternatively, the action of carbon materials is powerfully dependent on their physic-chemical properties for instance specific surface area, porosity and the existence of heteroatoms in the carbon structure. Literature survey has revealed^12–14^, that doping of heteroatoms for example, nitrogen atoms in the carbon structure is able to improve the electric and chemical properties of carbon substances and extend their uses. The N-rich porous carbons (NPCs) shows great affinity for metal ions because of the amine functional groups on its structures. NPCs as a novel porous material, have lately developed as multipurpose stages for the heterogeneous catalysis^15^. The structure of NPCs has a great number of enduring pores shaped by widespread chemical cross-linkage to deliver a great specific surface area. To obtain NPCs the carbonization of substrates involving heteroatom has been proposed. Nevertheless, the control of the essential part of properties of the carbon substance via simple carbonization is perplexing, for example relating templates. Nevertheless, the great number of the templates are synthetic and their utilization is expensive and delays the synthetic course of carbon materials. In addition, these catalysts continuously achieved with a considerable reduction in activity due to the metal leaching. Furthermore, the metal agglomeration and supporting materials failure typically happened during the process of catalyst separation, recovery and reusing. This inadequacy could be better by applying suitable support which being able to have strong coordination and unbending structure. More outstandingly, the attendance of these groups emerge the option of modifications with more desirable properties or the synthesis of heterogeneous catalysts^16,17^.

In the continuation of our interest in revealing the usefulness of nitrogen doped mesoporous carbons^18–21^, in this investigation, we purpose to apply γ-Fe_2_O_3_ hollow sphere as a magnetic support for the synthesis of low density and tuning the textural aspects of N-doped carbons. In this work, melamine-based mesoporous polymer network as a carbon precursor was created in the presence of amine-functionalized γ-Fe_2_O_3_ hollow sphere covered with glucose-derived carbon. Consequently, the obtained nanomagnetic N-rich polymer was employed for the immobilization of Pd nanoparticles. To adapt the surface properties and presenting more porosity, N-rich polymer was next carbonized to give the desired palladated mesoporous carbon. The obtained catalyst, Pd@h-Fe_2_O_3_@C, showed outstanding catalytic activity, selectivity and reusability in two important reactions namely, the Suzuki and Sonogashira cross coupling reactions. Indeed by employing and comparing several control catalysts involving, Pd@h-Fe_2_O_3_, Pd@h-Fe_2_O_3_@MRF, Pd@h-Fe_2_O_3_@glu, Pd@h-Fe_2_O_3_@glu-RF, Pd@h-Fe_2_O_3_@glu-MRF, Pd@h-Fe_2_O_3_@MRF-C, Pd@h-Fe_2_O_3_@glu-RF-C of their Pd loading, leaching, specific surface area and catalytic activity with those of the catalyst, the key features and roles of carbonization the catalytic activity of polymer were examined.

## Result and discussion

### Catalyst characterization

First, morphological characteristics of the Pd@h-Fe_2_O_3_@C including shape, size, and particle distribution envisioned by TEM, high resolution transmission electron microscopy (HRTEM) and selected area electron diffraction (SAED) pattern. TEM images displayed hollow-spherical morphology albeit with some amount of agglomeration and the diameter of the Pd nanoparticle was about 17 nm (Fig. 2A,C). Moreover, it is clearly that the spheres are surrounded with gray coverage, confirming the successful incorporation of glucose and organic layer in the structure of the catalyst^22^. As can be seen, the HRTEM image displays the crystalline lattice structure inside the Pd NPs. The lattice fringe spacing was measured to be 0.20–0.28 nm (Fig. 2D,E), which can be attributed to the Pd (111) plane. Figure 2F represents its SAED pattern, indicating different planes, which matches with the XRD planes. From the histogram, the mean diameter was found that ∼16.1 nm with a standard deviation of 4 nm. The average diameter of the Pd nanoparticles should be equal to the distance (20 nm), estimated above. Furthermore, the average diameter size of Palladium NPs was calculated by employing Debyee-Scherrer equation, being about 19.5 nm. The variation in the mean diameter from one agglomerate to another is acceptable and may be attributed to the blurring of boundaries in the 3D structures. As visible, our synthesis method resulted in a selective deposition of the external Pd layer at the surface of the magnetic composite.

**Figure 1:**
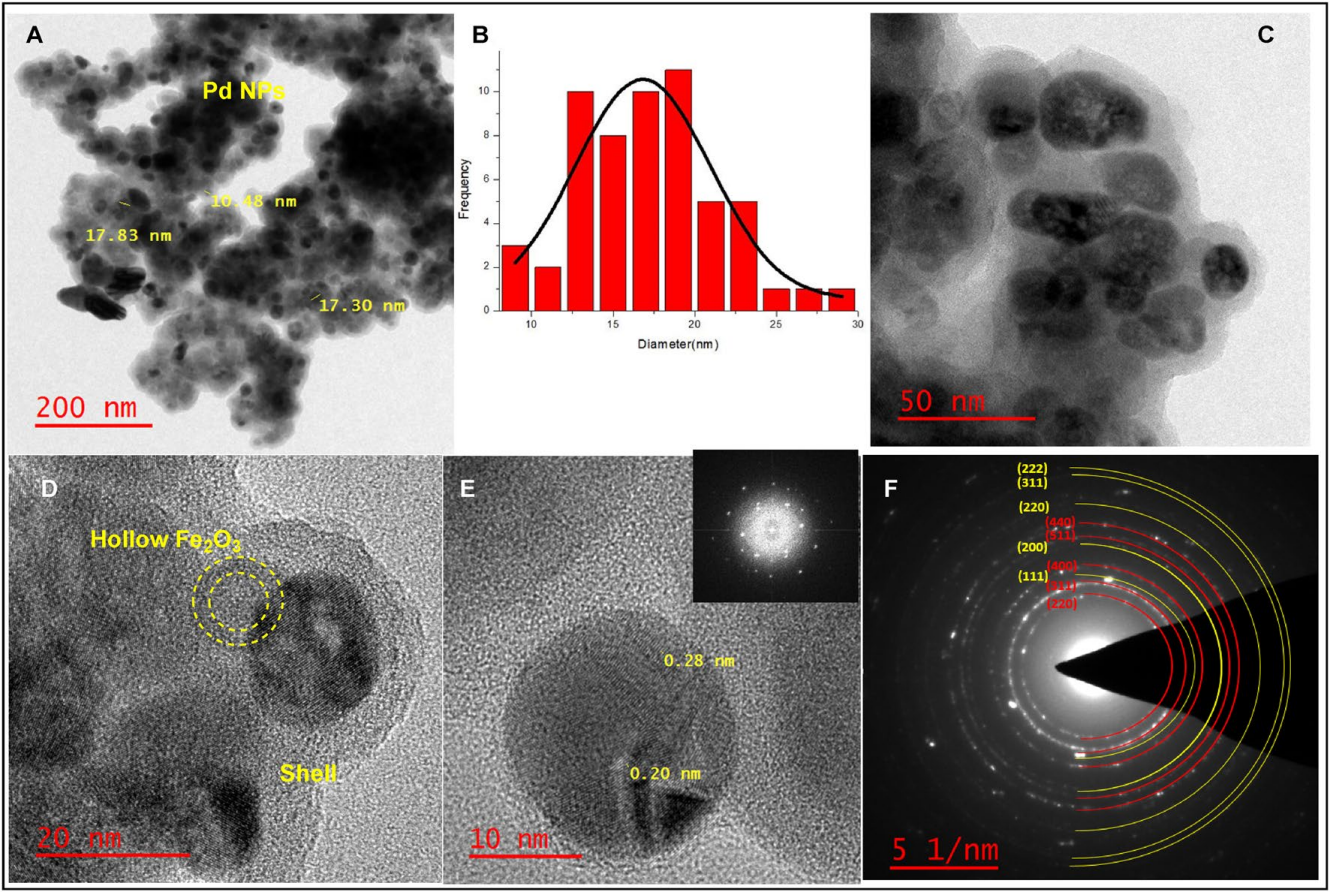
**(A,C)** TEM images of Pd@h-Fe_2_O_3_@C, **(B)** size distribution histogram of Pd NPs, **(D,E)** HRTEM images of Pd NPs and **(F)** SAED pattern of Pd@h-Fe_2_O_3_@C.

**Figure 2:**
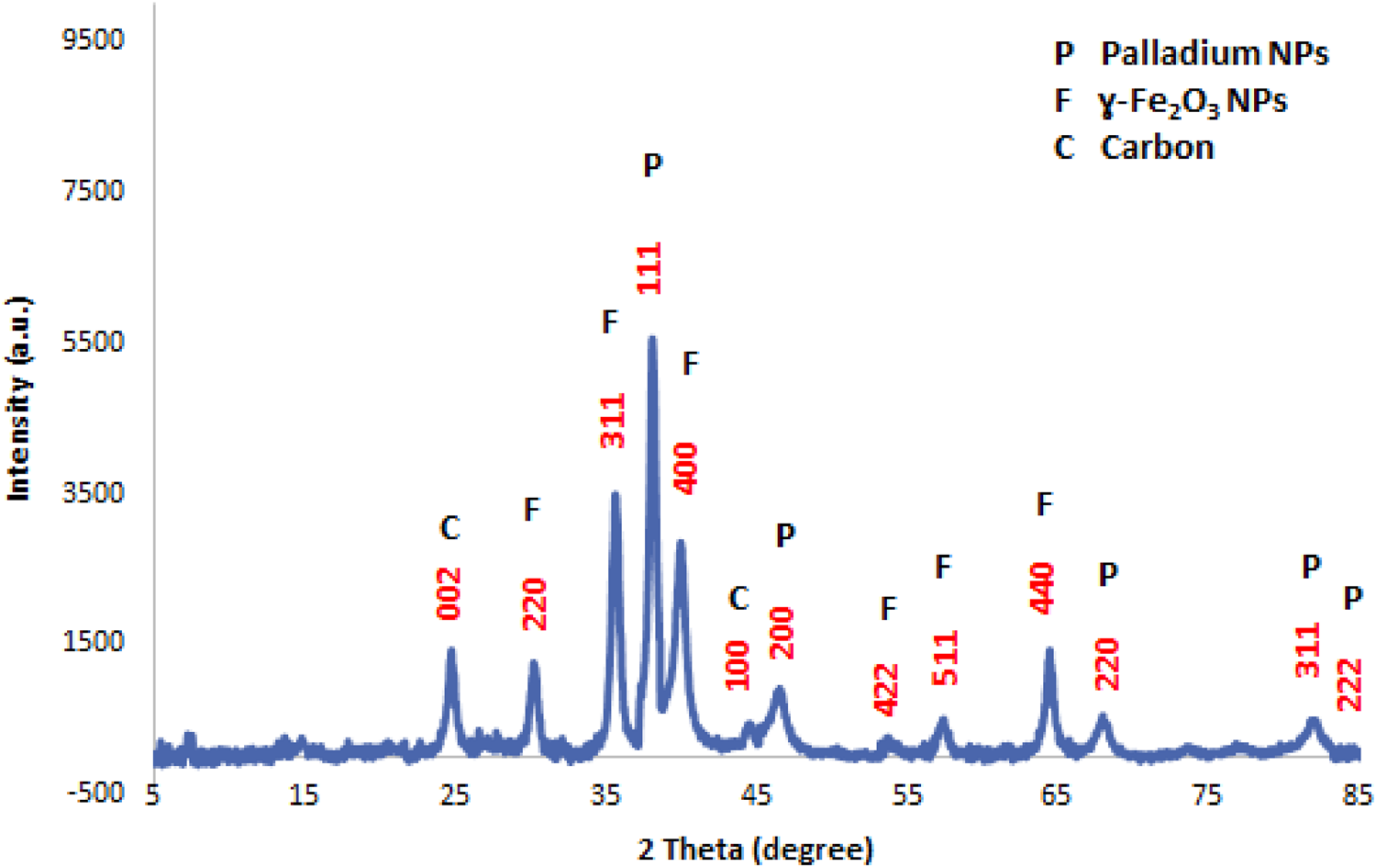
XRD pattern of Pd@h-Fe_2_O_3_@C.

Next, FTIR spectroscopy was applied to verify the formation of Pd@h-Fe_2_O_3_@C and the compounds prepared in the course of synthesis of Pd@h-Fe_2_O_3_@C. The FTIR spectra of the Pd@h-Fe_2_O_3_@C (a), h-Fe_2_O_3_@glu-MFR (b) and pure glucose (c) are depicted in Supplementary, Fig. S1. This spectrum (a) clearly showed the characteristic bands of h-Fe_2_O_3_, i.e. the strong absorption bands at 470–590 cm^−1^, which can be assigned to Fe–O stretching and the strong band at 3463 cm^−1^, which can be attributed to –OH groups^11^. The characteristic bands of glucose (c) can be listed as the bands at 3411, 2943 and 1461 cm^−1^ that can be assigned to the -OH functionality and –CH_2_ stretching. The FTIR spectrum of h-Fe_2_O_3_@glu-MFR (b) exhibited the characteristic bands at 1604, 2850, 2942 and 3406 cm^−1^ can be due to the C=N, –CH_2_ stretching and -OH functionality, which confirms the conjugation of organic layer. Moreover, the FTIR spectrum (b) showed the characteristic bands of magnetic core (h-Fe_2_O_3_), implying that the magnetic core preserved its structure upon functionalization processes. The FTIR spectrum (a) of Pd@h-Fe_2_O_3_@C is significantly distinguished from others and the intensity of the characteristic bands became very weak. This observation of some of characteristic bands is quite expectable and can be due to the high temperature thermal treatment and carbon generation.

The structure and formation of γ-Fe_2_O_3_ hollow sphere as well as palladium NPs was also studied by X-ray diffraction pattern of the catalyst (Fig. 2). As shown, the characteristic peaks of γ-Fe_2_O_3_ hollow sphere were appeared at 2*θ* = 30.6° {220}, 34.9° {311}, 43.5° {400}, 54.1° {422}, 57.5° {511}, 63.5° {440} and 74.2° {533} (labelled as F) can indicate the typical cubic structure hematite (JCPDS card No. 39–1346)^11^. In the XRD pattern of Pd@h-Fe_2_O_3_@C, the peaks labelled as P are characteristic peaks of palladium NPs at 2*θ* = 40.0°, 46.6°, 68.5°, 82.3° and 87.1° (JCPDS, No.46-1043) that can be assigned to the {111}, {200}, {220}, {311} and {222} planes^23^. In the XRD pattern of Pd@h-Fe_2_O_3_@C, three bands was observed that can be assigned to the carbon material. Precisely, the bands at 2*θ* = 25.3°, 45.0° 81.7° that can be indexed to {002}, {100} and {110} crystal planes respectively^24^, belonging to the hexagonal graphite (JCPDS card No. 41-1487)^25^. It is worth mentioning that the characteristic bands at 2*θ* = 81.7° was overlapped with that of Palladium NPs.

Raman spectroscopy was also applied for the characterization of the catalyst (see Supplementary Fig. S2). The Raman spectrum of Pd@h-Fe_2_O_3_@C exhibited two bands at 1357 (D-band) and 1601 cm^−1^ (G-band), related to graphitic carbon, can confirm the graphitic nature of the catalyst. In greater detail, the D-band is indicative of the sp^3^ configuration that can be attributed to presence of intrinsic defects and the G-band can be attributed to the graphitic carbon^26^. In this line, the I_D_/I_G_ was calculated and measured to be 0.83, can confirm disordered graphitic structures or highly defective^27^.

To elucidate whether decoration of the surface of h-Fe_2_O_3_ with carbon shell and palladium NPs can alter the magnetic property, Pd@h-Fe_2_O_3_@C was studied by room temperature vibrating sample magnetometer (VSM), and its magnetic features compared with that of h-Fe_2_O_3_ (see Supplementary Fig. S3). As obvious, the maximum saturation magnetization (*Ms*) values of h-Fe_2_O_3_ and Pd@h-Fe_2_O_3_@C were found to be 45.1 and 33.1 emu/g, respectively. Clearly, the *M*_s_ value of the hollow Fe_2_O_3_ nano sphere (45.1 emu/g) is higher than that of the catalyst which may be due to the incorporation of non-magnetic compounds and immobilization of palladium NPs on the surface of magnetic core. However, the hysteresis loops of the catalyst showed a paramagnetic behaviour without aggregation that can be easily separated from the reaction mixture using an external magnetic force.

In Supplementary Fig. S4, the thermal stability of the (a) h-Fe_2_O_3_@glu-MFR, (b) h-Fe_2_O_3_ and (c) Pd@h-Fe_2_O_3_@C were recorded using TG analysis and were compared. As shown, the h-Fe_2_O_3_ possessed high thermal stability and exhibited only the weight loss below 200 °C that is representative of loss of water. The comparison of h-Fe_2_O_3_@glu-MFR with that of h-Fe_2_O_3_ indicates that apart from the weight losses of magnetic core, an major weight loss at 450 °C can be detected that can be attributed to the degradation of organic layer. More detailed, the weight loss of about 19% can be due to the decomposition of MRF as an organic motif that indicates the successful formation of MRF in the structure of the catalyst. Considering the thermo gram of Pd@h-Fe_2_O_3_@C, it can be concluded that this sample exhibited significantly higher thermal stability compared to that of others, confirming the successful carbonization.

In the following, the effect of the carbonization of organic shell on the textural properties of the catalyst was studied. To this purpose, the N_2_ adsorption–desorption isotherms of Pd@h-Fe_2_O_3_@C and h-Fe_2_O_3_ were recorded and depicted in Supplementary Fig. S5. As shown, the isotherms of two samples are distinguished. The shape of h-Fe_2_O_3_ exhibited type II isotherm, while Pd@h-Fe_2_O_3_@C showed type IV with H3 hysteresis loops^24^. To further verify this issue, the specific surface area of two samples were calculated and compared. The specific surface area of the catalyst was calculated to be 426 m^2^g^−1^ which was higher than that of h-Fe_2_O_3_ (53 m^2^g^−1^). This can indicate the porous nature of Pd@h-Fe_2_O_3_@C. The total pore volume of two samples were also compared (see Supplementary Fig. S5). More precisely, this value for Pd@h-Fe_2_O_3_@C was much higher than that of h-Fe_2_O_3_, indicating the carbonization of organic layer resulted in the formation of pores. Moreover, the pore size distribution curves of the h-Fe_2_O_3_ and Pd@h-Fe_2_O_3_@C were obtained by the BJH method using the pore volumes in the measurement of N_2_ desorption isotherms. As is evident from pore size distribution result of Pd@h-Fe_2_O_3_@C, two types of pores with mesoporous (2 nm) and micropores (8.9 and 11 nm) were clear. Nevertheless, compared to the Pd@h-Fe_2_O_3_@C, the microporous size uniformity has increased and appeared at 8.9 and 11 nm, presumably due to the carbonization of organic layer.

Finally, ICP-AES was exploited for measuring the content of Fe and palladium NPs in the catalyst. To prepare the sample for the analysis, a known quantity of Pd@h-Fe_2_O_3_@C was digested in a mixture of concentrated HCl and HNO_3_ solution. The obtained extract was analysed and the results confirm that the loading of Fe and palladium were 2.47 and 0.075 mmol g^−1^, respectively.

### Investigation of the catalytic activity

To assess the catalytic activity of this heterogeneous system, the Pd@h-Fe_2_O_3_@C was utilized as a recyclable catalyst in Suzuki coupling reaction. Initially, the reaction of iodobenzene **1** and phenylboric acid **2** was selected as a model substrate. The reaction was conducted in the present of Na_2_CO_3_ and Pd@h-Fe_2_O_3_@C (0.5 mol%) at 75 ℃ in EtOH condition. After 1 h, the nature of the reaction mixture quickly changed to a dark viscous solid. After purification by column chromatography, the biphenyl **3a** was afford in 35% yield. Ultimately, the experimental results established that the biphenyl could achieved with 95% yield when the model reaction was carried out in the present of Pd@h-Fe_2_O_3_@C (0.5 mol%) with Na_2_CO_3_ at 75 ℃ in water/EtOH.

Further, to determine its scope by applying various aryl halides, a range of reactions was carried out under the optimal reaction conditions. As shown in Table 1, a series of aryl halides with electron-donating and electron-withdrawing group and phenyl boronic acid were used to the reaction under optimized conditions. Eventually, the Pd@h-Fe_2_O_3_@C efficiently catalyzed the coupling reaction between aryl halides with phenylboronic acid and biphenyls were attained in high to excellent yields after purification. In detail, the electronic effect of the substituents was generally found have no considerable influence since aryl iodides bearing donor- and acceptor substituents reacted with phenylboronic acid to afford the expected coupled products in excellent yields. Worthy to mention that, when iodobenzene was substituted by bromobenzene, the reactions needed longer reaction times for being completed. Notably, the scope of this methodology was found not to be operative to chlorobenzene.

**Table 1:** Pd@h-Fe_2_O_3_@C catalyzed Suzuki–Miyaura reaction of various aryl halides with boronic acid.


Product	X	R_1_	Time (min)	Yield^a^ (%)
3a	I	H	60	95
3b	I	4-Me	80	80
3c	I	4-OMe	55	90
3d	I	4-COMe	70	85
3e	I	4-NO_2_	60	80
3f	Br	H	125	70
3g	Br	4-Me	105	77
3h	Br	4-NH_2_	140	80
3i	Cl	H	165	63
3j	Cl	4-NH_2_	150	80
3k	Cl	2-NO_2_	130	82
3l	Cl	4-Me	170	75

Reaction condition: aryl halides (1.0 mmol), boronic acid (1.2 mmol), Pd@h-Fe_2_O_3_@C (0.5 mol%), Na_2_CO_3_ (2.0 mmol) in water:EtOH (5.0 mL) at 75 °C.

^a^Isolated yields.

Furthermore, the performance of Pd@h-Fe_2_O_3_@C catalyst was tested for the Sonogashira coupling reaction using bromobenzene **1** and phenylacetylene **4** as a model substrate. As shown in Supplementary Table S2, the optimization reaction conditions were investigated in different solvents, temperatures, bases, and in the presence of various amount of the Pd@h-Fe_2_O_3_@C catalyst. It was found that, when the model substrate was applied in the presence of Na_2_CO_3_ (2.0 mmol) at 50 ℃ using Pd@h-Fe_2_O_3_@C (0.35 mol%) as catalyst, the yield of product could reach 98% after separation. Using the optimized reaction conditions, the scope and generality of this method were exemplified in the reaction of aryl halides **1** and phenyl/aliphatic acetylenes **4** using Pd@h-Fe_2_O_3_@C catalyst, and the outcomes are presented in Table 2. The cross-couplings of phenyl/aliphatic acetylenes with aryl iodides bearing electron donating groups, –OMe, –Me and –COMe gave products in satisfactory yields. Moreover, arylbromides and chlorides are efficiently reacted as substrates in this process, though, the reaction of aryl chlorides needed longer reaction times for being completed. Noticeably, propargylalcohol as an aliphatic acetylene was also fruitfully coupled to arylhalides with satisfactory yields. Nevertheless, the highest yields were obtained for aryl acetylene. Noteworthy, all compounds are known and some were identified by comparing physical properties through FTIR and melting point analyses.

**Table 2:** Pd@h-Fe_2_O_3_@C catalyzed Sonogashira reaction of various aryl halides with terminal alkynes.


Product	X	R^1^	R^2^	Time (h:min)	Yield^a^ (%)
5a	I	H	C_6_H_5_	00:35	93
5b	I	4-Me	C_6_H_5_	00:45	98
5c	I	4-OMe	C_6_H_5_	00:25	95
5d	I	4-COMe	C_6_H_5_	00:40	90
5e	I	H	CH_2_OH	01:00	88
5f	I	4-Me	CH_2_OH	01:15	85
5g	Br	H	C_6_H_5_	01:20	98
5h	Br	H	CH_2_OH	01:50	78
5i	Br	4-Me	C_6_H_5_	02:10	65
5j	Br	4-NH_2_	C_6_H_5_	02:45	72
5k	Cl	H	C_6_H_5_	03:00	80
5l	Cl	H	CH_2_OH	03:45	70
5m	Cl	4-NH_2_	C_6_H_5_	05:10	55
5n	Cl	2-NO_2_	C_6_H_5_	04:55	55
5o	Cl	4-Me	C_6_H_5_	05:15	58

Reaction condition: aryl halides (1.0 mmol), terminal alkynes (1.2 mmol), Pd@h-Fe_2_O_3_@C (0.35 mol%), Na_2_CO_3_ (2.0 mmol) in H_2_O (5.0 mL) at 50 ˚C.

^a^Isolated yields.

Next, the chemical state-catalytic activity relationship was studied. Initially, the result of loading of Pd on the support was studied. In this regard, apart from the catalyst two more samples with different loading of Pd, i.e. Pd@h-Fe_2_O_3_@C (Pd 2 and 3 wt%). The outcomes showed that the loading of Pd meaningfully influence the catalytic activity and use of lower content of Pd is more effective. Then, the influence of each component in the structure of the catalyst to the catalysis was studied. For this purpose, several control catalysts, including, Pd@h-Fe_2_O_3_, Pd@h-Fe_2_O_3_@glu, Pd@h-Fe_2_O_3_@glu-MRF, Pd@h-Fe_2_O_3_@glu-RF, Pd@h-Fe_2_O_3_@glu-RF-C and Pd@h-Fe_2_O_3_@C were prepared (see experimental section). As shown in Table S3, entry 1, Pd@h-Fe_2_O_3_ is not an active catalyst and the product was obtained only 35%. Then, it was studied whether introducing of Glu shell is able to improve the catalytic activity. In this line, Pd@h-Fe_2_O_3_@glu was provided and its catalytic activity was examined and the product was obtained with 50% yield (Table S3, entry 4). This results approved that Glu can somewhat improve the catalytic activity. For further revealing the key role of Glu in the catalysis, two control catalysts, Pd@h-Fe_2_O_3_@MRF and Pd@h-Fe_2_O_3_@MRF-C was prepared, in which the Glu was not present in the structure of the catalyst and the resorcinol–formaldehyde-melamine polymer was adjusted on the surface of h-Fe_2_O_3_ and subsequently palladated and carbonized (Table S3, entries 2 and 3). Based on the comparison of the results, the contribution of Glu was confirmed. In details, donor- and acceptor substituted it was found that Glu component not only influenced the catalytic activity, but also improved the separation and reusability of the catalyst. More exactly, the separation of the catalyst was simpler and more efficient than that of Pd@h-Fe_2_O_3_@MRF and Pd@h-Fe_2_O_3_@MRF-C, while, the ICP examination of these samples approved the higher loading of Pd NPs in the catalyst. Approving the role of melamine as a nitrogen source, the effect of N-precursor by creating another control samples, Pd@h-Fe_2_O_3_@glu-RF, Pd@h-Fe_2_O_3_@glu-RF-C in which melamine was omitted in the structure of polymer was clarified and compared with Pd@h-Fe_2_O_3_@glu-MRF and catalyst. As tabulated, melamine as an N-rich precursor has the ability to produce product with the highest catalytic activity (Table S3, entries 5–8). To elucidate the effect of polymer's type on the structure of the catalyst and Pd loading, the specific surface area of these samples were compared and showed that this value decreased in the following order: Pd@h-Fe_2_O_3_@C (426 m^2^g^−1^) > Pd@h-Fe_2_O_3_@glu-RF-C (156 m^2^g^−1^) > Pd@h-Fe_2_O_3_@glu-MRF (42 m^2^g^−1^) > Pd@h-Fe_2_O_3_@glu-RF (33 m^2^g^−1^). By these results the effect of the N-rich carbon precursor on the content and specific area of the carbon coated h-Fe_2_O_3_ was confirmed. Further, the effects of composite components on the loading and leaching of Pd NPs in catalyst as well as control samples were measured via ICP analysis and studied, Table S3. As tabulated, the order of loading and leaching of Pd increased in the following order: Pd@h-Fe_2_O_3_ > Pd@h-Fe_2_O_3_@MRF > Pd@h-Fe_2_O_3_@glu > Pd@h-Fe_2_O_3_@glu-RF > Pd@h-Fe_2_O_3_@glu-MRF > Pd@h-Fe_2_O_3_@MRF-C > Pd@h-Fe_2_O_3_@glu-RF-C > Pd@h-Fe_2_O_3_@C. The lowest loading of Pd was observed in Pd@h-Fe_2_O_3_ sample which can be due to the absence of strong chemical interaction between Pd NPs and h-Fe_2_O_3_ surface. It is worth mentioning that the low catalytic activity of this sample can be allocated to the low Pd loading. The loading of Pd in Pd@h-Fe_2_O_3_@MRF, Pd@h-Fe_2_O_3_@glu, Pd@h-Fe_2_O_3_@glu-RF and Pd@h-Fe_2_O_3_@glu-MRF samples compared to that of Pd@h-Fe_2_O_3_ can confirm that the presence of carbon shell could increase Pd immobilization. Nevertheless, the Pd leaching of this sample was relatively high. Pd@h-Fe_2_O_3_@MRF-C and Pd@h-Fe_2_O_3_@glu-RF-C that possessed carbon shell, the Pd anchoring were further enhanced compared to Pd@h-Fe_2_O_3_@MRF and Pd@h-Fe_2_O_3_@glu-RF, indicating the encouraging effect of carbonization of polymeric shell on immobilization of Pd NPs. Furthermore, the Pd leaching in samples were repressed compared to that of Pd@h-Fe_2_O_3_@glu-MRF and Pd@h-Fe_2_O_3_@glu-RF. In Pd@h-Fe_2_O_3_@C, the loading of Pd was increased compared to that of Pd@h-Fe_2_O_3_@glu-MRF, representing that Pd anchoring on carbonized sample was most better that un-carbonized one.

Furthermore, the efficiency of this protocol and the catalytic performance of Pd@h-Fe_2_O_3_@C for catalyzing the Suzuki and Sonogashira model reactions were compared with those of some previously reported catalytic methodologies to disclose the merits of this procedure (Table S4). As obvious, Pd@h-Fe_2_O_3_@C resulted in desired product in higher or comparative yields. However, compared to all cases, Pd@h-Fe_2_O_3_@C led to the product in shorter reaction time. Moreover, this protocol does not required any harsh reaction condition, inert atmosphere or toxic solvent^28–33^.

### Catalyst recyclability

**Figure 3:**
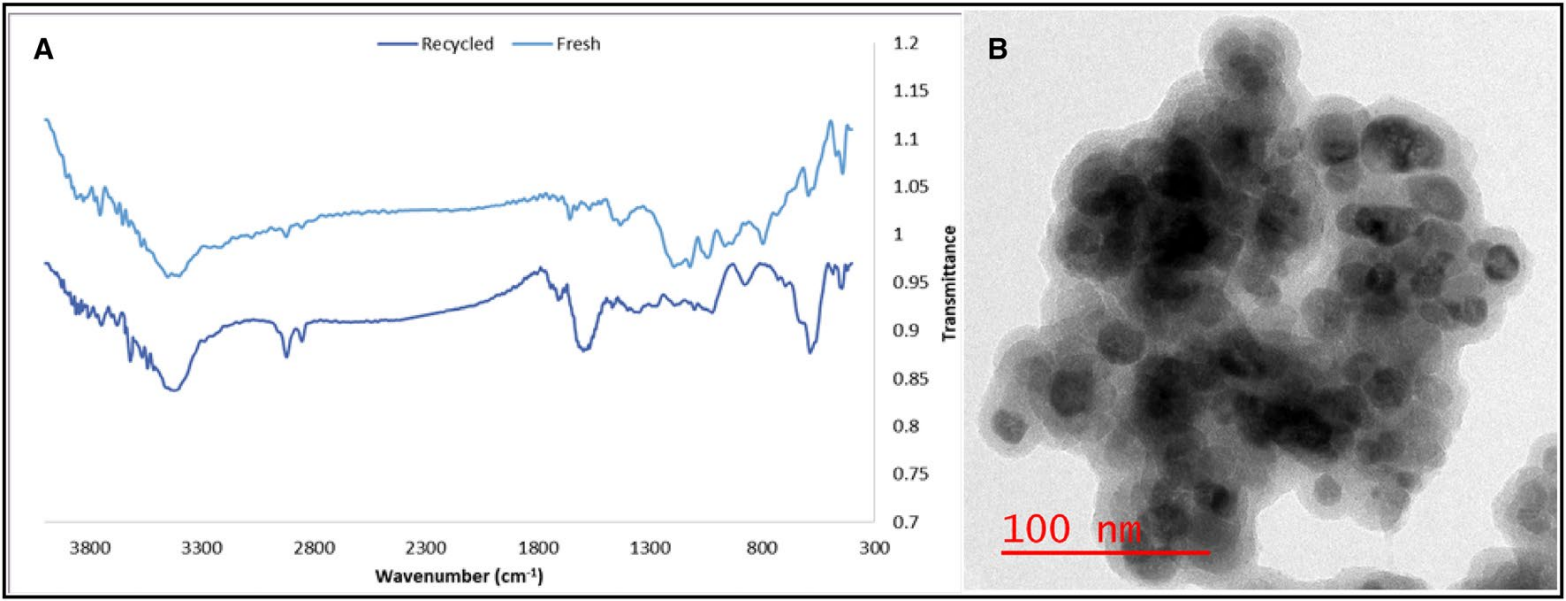
The FTIR spectra (A) of fresh and recycled Pd@h-Fe_2_O_3_@C catalyst and TEM image (B) of the recycled catalyst after five runs.

To explain whether Pd@h-Fe_2_O_3_@C can be considered as a reusable catalyst, the recycling of that catalyst was carried out for the Sonogashira model reaction and the results exhibited in Supplementary as Fig. S6. As shown, the catalyst was subjected to five successive runs. Noteworthy, up to the fourth reaction run, only a reasonable decrease was detected, subsequently a more obvious loss of activity after the fifth run and the yield of product reached to 44%. The recycling could have also been hampered by partial structural damage to support. There was also observed increase in mass of the solid due to the presence of the catalyst, solid base, and salt making reproducibility difficult after every run. In addition, washing with water after separation reduced the activity of the catalysts, because the pore size of the surface of mesoporous carbon shell is smaller than palladium nanoparticles. Filtering also leads to loss of some Pd NPs as an active sites. However, a hot filtration test proved the heterogeneous nature of the supported catalyst and without a measurable homogenous contribution. Table S3 gives the results of catalyst leaching in the Sonogashira coupling reaction of aryl halide and phenyl acetylene. According to the ICP-AES analysis, the Pd content of the heterogeneous catalyst was determined to be 0.075 mmol/g^−1^. The percentage of Pd leaching/Pd loading was around 4.6%. The soluble leached Pd could likely be responsible for catalysis in this reaction. Pd leaching correlates significantly with the progress of the reaction, the nature of the starting materials and products, solvent, base, and atmosphere. These results agree with Shmidt and Mametova’s observation of the oxidative attack of the halide to the metal crystallites, yielding directly Pd(II) in solution^34^. Generally, the metal ions are probably first reduced in the presence of base, which causes some leaching, which is followed by oxidative addition of aryl halide and substantial leaching^35^. To consider the effects of reusing on the morphology of the catalyst, FTIR spectra and TEM analysis of the recycled catalyst were recorded and compared with that of the fresh catalyst. The FTIR spectrum of the recycled Pd@h-Fe_2_O_3_@C demonstrated the specific bands of the fresh Pd@h-Fe_2_O_3_@C (Fig. 3a). Nevertheless, some difference between two spectra were observed, that can be due to the disposition of organic substances on the surface of the catalyst. The TEM image of the recycled Pd@h-Fe_2_O_3_@C demonstrated some of the agglomerated nanoparticles, which can be due to the magnetic nature of nanoparticles. However, the spherical structure of the nanoparticles is preserved (Fig. 3b). Furthermore, the ICP-AES analysis of the filtrate exhibited Pd and Fe content 0.0031 and 0.19 mmol g^−1^, respectively.

## Methods

### Synthesis of the catalyst

#### Synthesis of nanomagnetic Fe_2_O_3_ hollow sphere (a)

The palladated-encapsulated h-Fe_2_O_3_ was fabricated through in situ polymerization. The procedure included the preparation of magnetic core, synthesis of prepolymer solution, and the formation of palladated-magnetic carbon shell. Nanomagnetic Fe_2_O_3_ hollow sphere as a magnetic core, was prepared through solvothermal method^11^. Initially, FeCl_3_.6H_2_O (5 mmol) was dissolved in 70 mL of ethylene glycol in a flask. Next, 30 mmol of sodium acetate trihydrate, 1.5 mmol of trisodium citrate dehydrate and urea (17 mmol) were added to the aforementioned solution. After stirring of the resultant mixture for 60 min, it was then transferred to the Teflon-lined stainless-steel autoclave (150 mL capacity) and kept at 220 °C overnight. Upon completion of the process, the reactor was cooled to ambient temperature and the brown product **1** was washed with EtOH/H_2_O and dried at 80 °C for 24 h in oven.

### Synthesis of h-Fe_2_O_3_@glu core–shell (b)

The resulting h-Fe_2_O_3_@glu core–shell was prepared by previous method with little modification, accordingly^36^. Initially, h-Fe_2_O_3_ (1 g) well dispersed in deionized water (40 mL) by using ultrasonic irradiation (power 100 W) for 15 min. Afterward, glucose (6 g) was introduced in the prepared magnetic suspension and the resulting mixture was transferred into a Teflon-lined stainless steel autoclave (150 mL). Then, the container was closed and maintained at 200 °C for one day. At the end of the hydrothermal treatment, the reactor was cooled down to room temperature and the product **2** was collected by a magnet bar, rinsed with EtOH, centrifuged for five times and dried in oven at 80 °C.

### Synthesis of h-Fe_2_O_3_@glu-Cl core shell (c)

In the next step, the surface of h-Fe_2_O_3_@glu was functionalized with (3-Chloropropyl)trimethoxysilane (CPTES), according to previous literature^37^, with a slight modification. For this, h-Fe_2_O_3_@glu (3 g) was dispersed by sonication in 100 mL dry toluene for 1 h, and then CPTES (3 mL) was injected dropwisely into the h-Fe_2_O_3_@glu suspension in the presence of Et_3_N (3 mL). The obtained mixture was heated and stirred continuously under reflux condition at 110 ◦C, for 24 h under Argon atmosphere. The mixture was cooled to room temperature and h-Fe_2_O_3_@glu-Cl, **3**, was magnetically collected and washed with toluene, and then dried under air for 12 h.

### Synthesis of h-Fe_2_O_3_@glu-MFR (d)

The pre-polymer solution was prepared by mixing formaldehyde (10 mL) and melamine (2 g) in distilled water (10 mL), according to previous method^38^. Next, the pH of the mixture was adjusted to 8.5–9.0 by adding TEA and stirred vigorously at 70 ◦C. When it became clearly transparent, another melamine (1 g) was added and stirred till it was dissolved completely. Similarly, water (10 mL) was added and stirred till the solution was transparent absolutely. On the other, h-Fe_2_O_3_@glu**-**Cl (0.5 g) well dispersed in water (20 mL) and stirred for half time. The magnetic suspension was adjusted to pH 4.5–5.0 with 15.0 wt.% acetic acid solution. The prepared solution was added into the magnetic suspension under stirring condition, dropwisely. After that, resorcinol was added to the prepared cross-linking with the M–F pre-polymer. After all of the pre-polymer was added, ammonium chloride (5 wt.%) as a nucleating agent was added into the solution, then it was stirred at 60 ◦C for 90 min. The pH of the mixture was adjusted to 9.0 with TEA solution, which completed the reaction. Then the resultant microcapsule **4** was magnetically collected, washed with EtOH until pH 7 was reached. The wet powders were dried in a vacuum oven at 100 ◦C overnight.

### Immobilization of palladium NPs on h-Fe_2_O_3_@glu-MFR (e)

Based on previous literature^20^ with little modification, to immobilize Palladium NPs on the h-Fe_2_O_3_@glu-MFR, a solution of Pd(OAc)_2_ (2 wt.%) in MeOH (10 mL) was added to the solution of suspended h-Fe_2_O_3_@glu-MFR (1 g) in toluene (50 mL) in a drop wise manner under stirring condition overnight. In the following, a solution of NaBH_**4**_ in MeOH (12 mL, 0.1 N) was added under inert atmosphere to the above-mentioned suspension. Subsequently, the resulting mixture was stirred for 7 h. The obtained Pd@h-Fe_2_O_3_@glu-MFR, was magnetically separated, washed with toluene and MeOH and dried in an oven at 80 °C for 12 h.

### Synthesis of Pd@h-Fe_2_O_3_@C (f)

**Figure 4:**
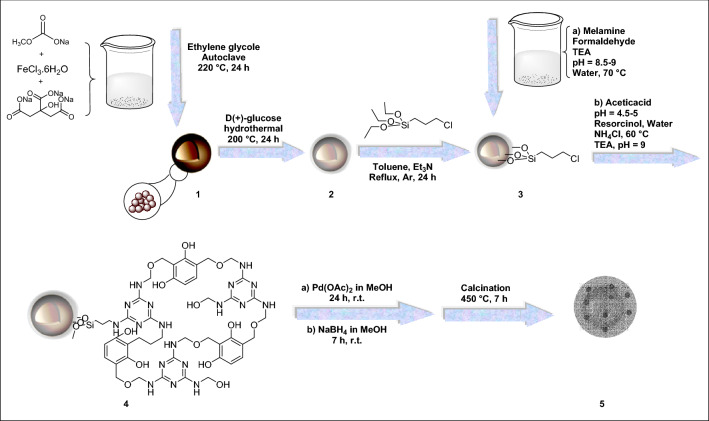
The possible formation process of Pd@h-Fe_2_O_3_@C catalyst.

The resulting Pd@h-Fe_2_O_3_@C was prepared by previous method^20^. To this propose, Pd@h-Fe_2_O_3_@glu-MFR (5 g) was placed in a quartz container and heated up to 450 °C for 1 h under argon flow and heating rate of 30 °C min^−1^. The sample was held at this temperature for 6 h. After cooling to room temperature, the product **5** (3 g) was washed and recovered as a black powder. The schematic processes of synthesis of the catalyst are depicted in Fig. 4.

### Catalytic activity

#### Sonogashira reaction

In a round bottom flask, aryl halide (1.0 mmol), acetylene (1.2 mmol) and Na_2_CO_3_ (2.0 mmol) were mixed in the presence of Pd@h-Fe_2_O_3_@C (0.35 mol%) as a catalyst in H_2_O (5.0 mL) and the resulting mixture was heated at 50 °C in an oil bath. The proceeding of the reaction was traced by TLC (n-hexane/ethyl acetate, 8:2). After finishing the reaction, Pd@h-Fe_2_O_3_@C was magnetically isolated, washed with EtOH repeatedly and dried in an oven at 60 °C for 6 h. Finally, the filtrate was extracted with diethyl ether for three times and then, the organic layer was washed with deionized water and dried over anhydrous Na_2_SO_4_ and purified using column chromatography over silica gel.

#### Suzuki reaction

A mixture of aryl halide (1.0 mmol), aryl boronic acid (1.2 mmol), Na_2_CO_3_ (2.0 mmol) and Pd@h-Fe_2_O_3_@C (0.5 mol%) was prepared in the mixture of water/EtOH (1:1, 5 mL) and was heated at 75 °C in an oil bath, subsequently. The solvent for monitoring reaction process by TLC was n-hexane/ethyl acetate, 7:3. Then, the mixture was cooled to ambient temperature and the catalyst was separated by a magnetic field, washed with ethanol for three times and dried in oven at 60 °C for 6 h. After that, he solvent was removed and the product was extracted with 10 mL of n-hexane. To achieve corresponding biaryls, the product was purified by column chromatography over silica gel.

### Characterizations of some products

#### 1,2-Diphenylethyne (5a)

White solid, mp: 61–63 °C lit.^39^; FT-IR (KBr): 3413, 3062, 2921, 1952, 1630, 1490, 1442, 1161, 916, 754.

#### 1-Methyl-4-(phenylethynyl)benzene (5b)^39^

White solid, m.p: 71–73 °C lit.^39^; FT-IR (KBr): 3552, 3474, 3414, 2958, 1626, 1500, 1261, 1098, 867, 751, 626.

#### 1-Methoxy-4-(phenylethynyl)benzene (5c)^39^

White solid, m.p: 65–66 °C lit.^39^; FT-IR (KBr): 3472, 3414, 2925, 1895, 1647, 1597, 1502, 1540, 1245, 1023, 821, 751, 685.

#### 3-Phenylprop-2-yn-1-ol (5e)

FT-IR (KBr): 3300, 3065, 2922, 2859, 2237, 1959, 1888, 1745, 1597, 1488, 1441, 1363, 1259, 1027, 955, 800, 756, 690, 571, 520.

## Conclusion

In summary, a magnetic composite *N*-doped mesoporous carbon with hollow morphology, Pd@h-Fe_2_O_3_@C, was prepared via coating of Glu carbon shell on h-Fe_2_O_3_, functionalization, and growth of MRF polymer, Pd immobilization followed by carbonization. The resulting core–shell established excellent catalytic activity for the C–C coupling reactions. The comparison of the catalytic activity of Pd@h-Fe_2_O_3_@C with that of some control samples including, Pd@h-Fe_2_O_3_, Pd@h-Fe_2_O_3_@MRF, Pd@h-Fe_2_O_3_@glu, Pd@h-Fe_2_O_3_@glu-RF, Pd@h-Fe_2_O_3_@glu-MRF, Pd@h-Fe_2_O_3_@MRF-C, Pd@h-Fe_2_O_3_@glu-RF-C, confirmed higher catalytic activity of the former. It can be stated that the carbonization of MRF polymer can intensely increase the specific surface area and pore volume, the greater catalytic activity of Pd@h-Fe_2_O_3_@C. The recyclability test also confirmed high recyclability of Pd@h-Fe_2_O_3_@C with slight Pd leaching and no Pd aggregation. Furthermore, the hot filtration test showed the heterogeneous nature of the catalysis.

## Supplementary Information

Supplementary Information.
