# Supplementary material for: Magnetic composite of γ-Fe2O3 hollow sphere and palladium doped nitrogen-rich mesoporous carbon as a recoverable catalyst for C–C coupling reactions
Source: Sci Rep. 2021 Nov 17;11:22409. doi: 10.1038/s41598-021-99679-x (PMC8599472; doi:10.1038/s41598-021-99679-x)
Supplement: Supplementary file 1 — Supplementary Information. [file 41598_2021_99679_MOESM1_ESM.docx]

**Supporting information**

**Magnetic Composite of γ-Fe_2_O_3_ Hollow Sphere and Palladium Doped Nitrogen-rich Mesoporous Carbon as a Recoverable Catalyst for C-C Coupling Reactions**

*Masoume Malmir ^a^, Majid M. Heravi ^a^*, Zahra Amiri ^a^, Kosar Kafshdarzadeh ^a^*

*^b^ Department of Physics & Chemistry, School of Science, Alzahra University, PO Box 1993891176, Vanak, Tehran, Iran.*

*Tel: (+98) 21-88041347. Fax: (+98) 21-88613935. E-mail:* [*mmh1331@yahoo.com*](mailto:mmh1331@yahoo.com) *and* [*mmheravi@alzahra.ac.ir*](mailto:mmheravi@alzahra.ac.ir)

- 1. **Experimental**

**1.1.1. Materials** **and instrument**s

All chemicals and reagents, including FeCl_3_∙6H_2_O, sodium acetate trihydrate, trisodium citrate dehydrate, TEOS, (3-Chloropropyl)trimethoxysilane, toluene, triethanolamine, acetone, ethanol, ethylene glycol (EG), melamine, formaldehyde, resorcinol, Pd(OAc)_2_ and KOH were analytical grade reagents, purchased from Sigma-Aldrich, and used without further purification. The progress of the organic reactions were monitored by TLC on commercial aluminum-backed plates of silica gel 60 F254, visualized, using ultraviolet light. ^1^H NMR and ^13^C NMR spectra were recorded on Bruker DRX-400 spectrometer at 400 and 100 MHz respectively. The catalyst characterization was performed by using various characterization techniques including, XRD, FTIR, BET, TGA, VSM, TEM and ICP-AES. Fourier transform infrared (FTIR) spectra were recorded on PERKIN-ELMER- Spectrum 65 instrument. Transmission electron microscope (TEM) images of the final catalyst were recorded using CM30300Kv field emission transmission electron microscope. X-ray diffraction (XRD) patterns were obtained by a Siemens D5000 85 diffractometer with Cu Kα radiation in 2θ range of 5-90°. Thermogravimetric analysis (TGA) under inert condition was performed at heating rate of 10 °C min^-1^ using METTLER TOLEDO instrument. BELSORP Mini II apparatus was applied for recording N_2_ adsorption-desorption isotherm of the catalyst (the sample preparation was carried out by preheating of the samples at 100 °C for 3 h). The used Raman spectrometer for analyzing the catalyst was TEKSAN-N1-541 Spectrum at k= 532 nm instrument. ICP analyzer used for measuring the Pd loading and leaching of the catalysts was Varian, Vista-pro. The facilities applied for the synthesis of the catalyst included, ultrasonic apparatus (Bandelin HD 3200 with output power between 100-200 W and tip TT13), Teflon-lined stainless steel autoclave (150 mL) and furnace equipped with inert gas.

**
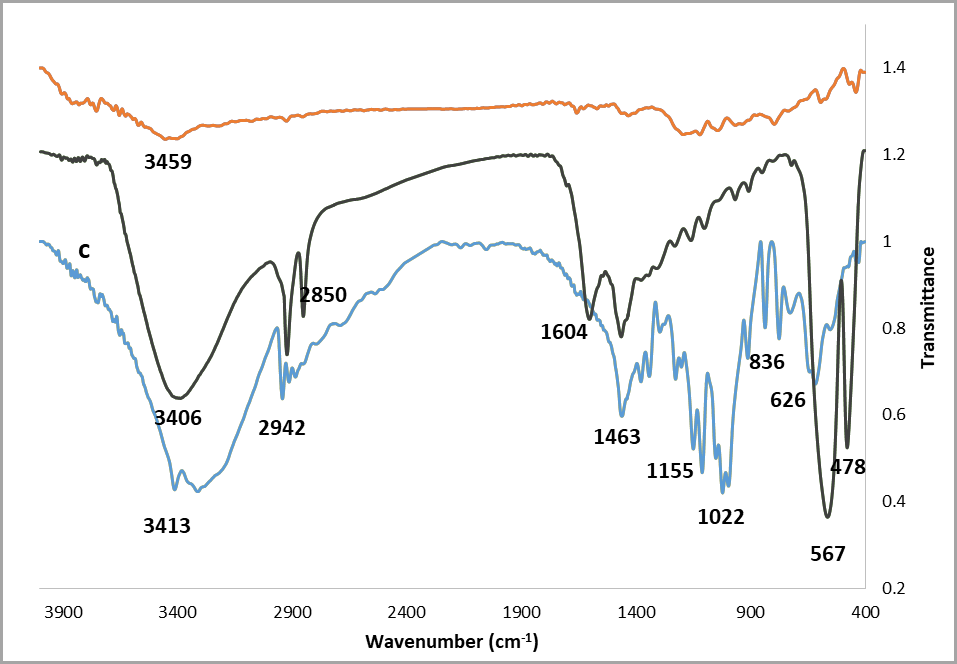
**

**Figure S1.** FTIR spectra of (a) Pd@h-Fe_2_O_3_@C, (b) h-Fe_2_O_3_@glu-MFR and (c) glucose.


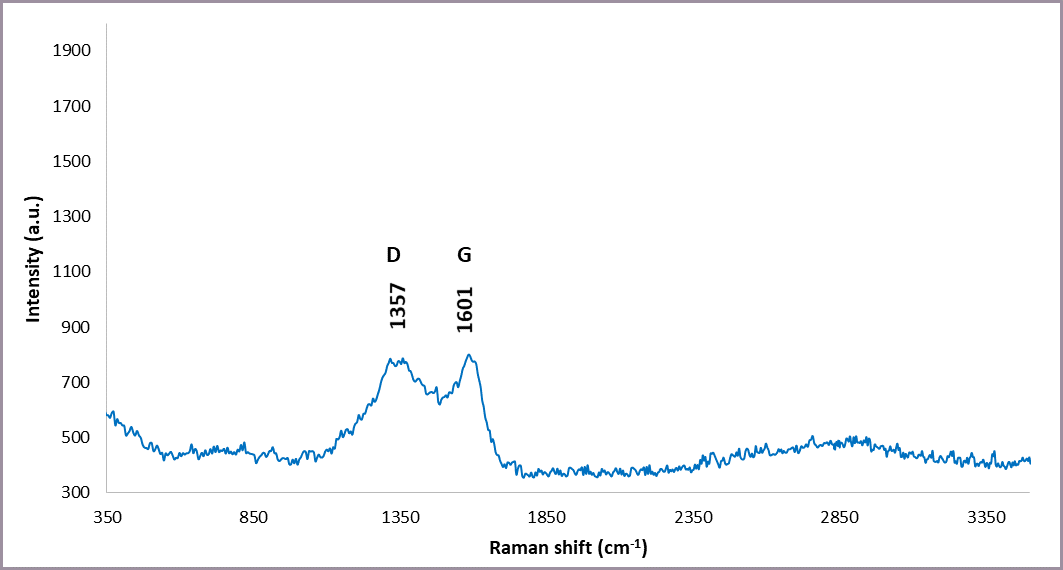


**Figure S2.** Raman spectrum of Pd@h-Fe_2_O_3_@C.


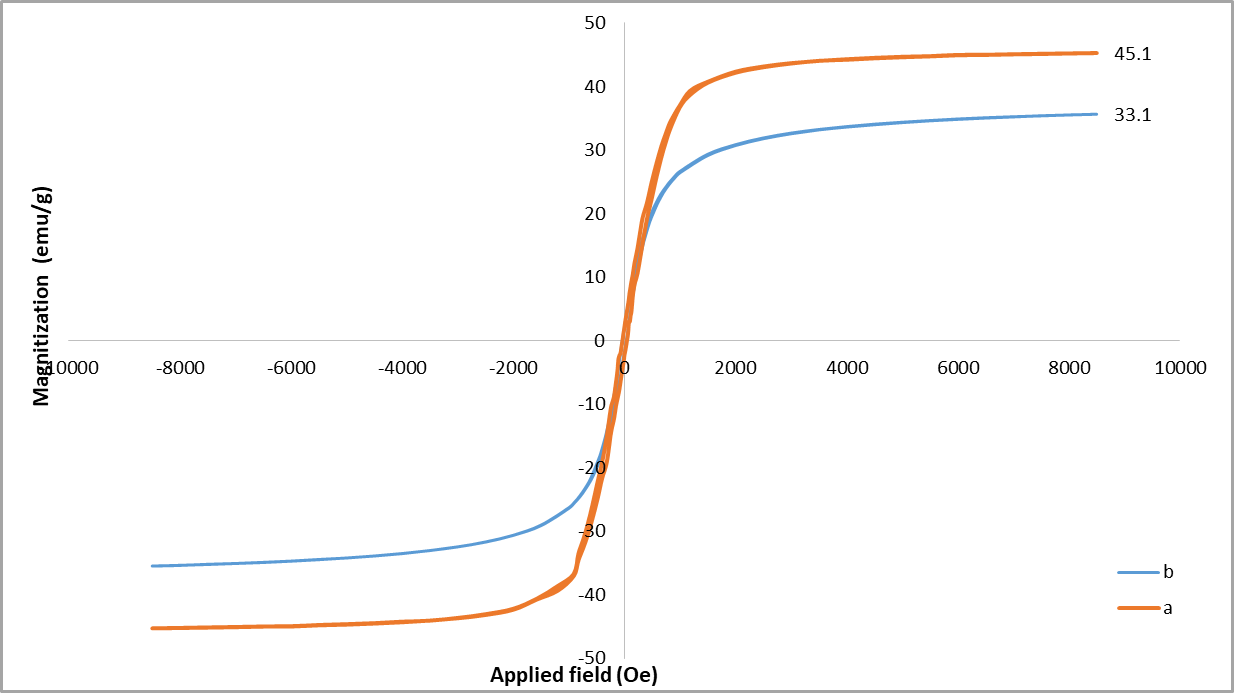


**Figure S3.** VSM analyses of (a) h-Fe_2_O_3_ and (b) Pd@h-Fe_2_O_3_@C.

**
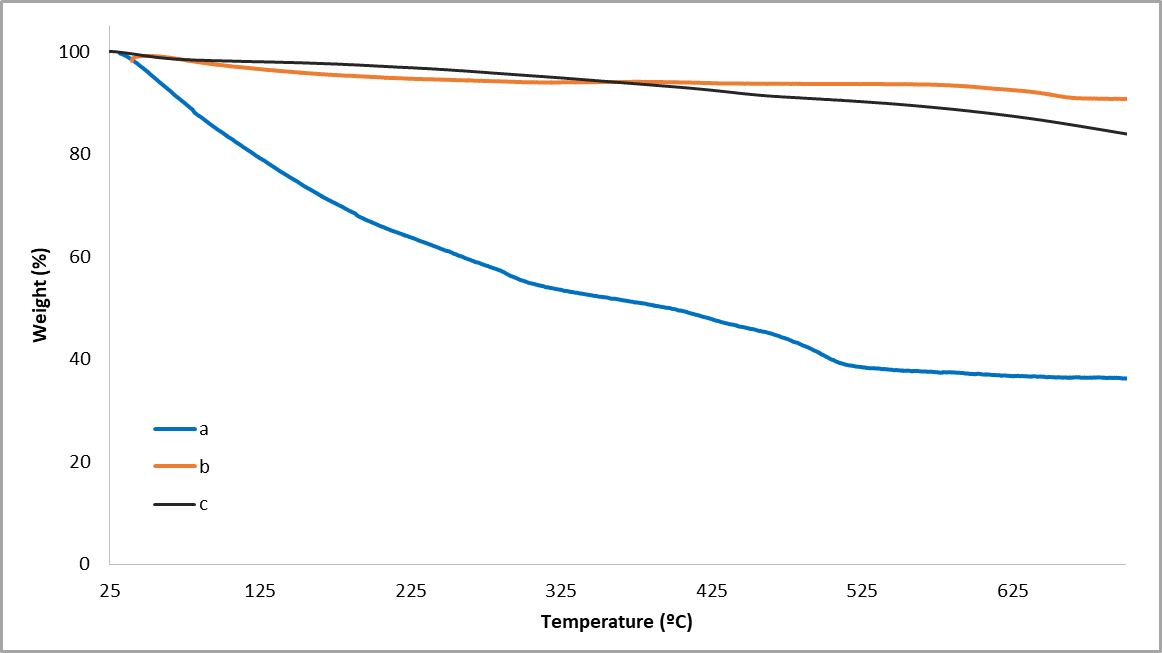
**

**Figure S4.** TG analyses of (a) h-Fe_2_O_3_@glu-MFR, (b) h-Fe_2_O_3_ and (c) Pd@h-Fe_2_O_3_@C.


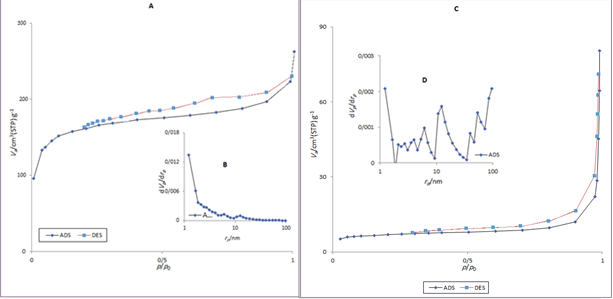


**Figure S5.** N_2_ adsorption-desorption and BJH plots of (A and B) Pd@h-Fe_2_O_3_@C and (C and D) h-Fe_2_O_3_.


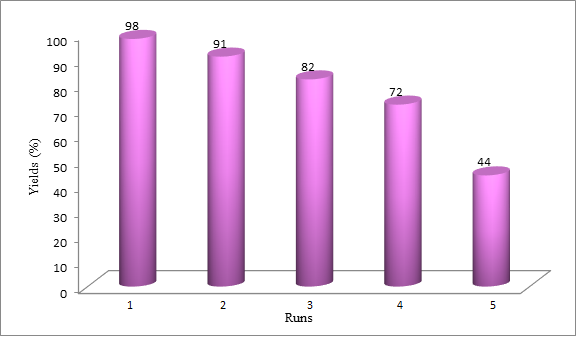


**Figure S6.** Recyclability of the Pd@h-Fe_2_O_3_@C catalyst in the Sonogashira coupling reaction.

| **Table S1**  Optimization of reaction conditions in the Suzuki coupling reaction of iodobenzene **1** with boronic acid **2^a^** | | | | | |
| --- | --- | --- | --- | --- | --- |
| **Entry** | **Loading of Catalyst**  **(mol%)** | **Condition**  **(solvent/ temperature °C)** | **Base** | **Time (min)** | **Yield^b^ (%)** |
| 1 | 0.5 | Water:EtOH/ r.t. | Na_2_CO_3_ | 60 | Trace |
| 2 | 0.5 | Water:EtOH/ 50 | Na_2_CO_3_ | 60 | 55 |
| 3 | 0.5 | Water:EtOH/ 75 | Na_2_CO_3_ | 60 | 95 |
| 4 | 0.5 | Water:EtOH/ 100 | Na_2_CO_3_ | 60 | 80 |
| 5 | 1 | Water:EtOH/ 75 | Na_2_CO_3_ | 60 | 80 |
| 6 | 1.5 | Water:EtOH/ 75 | Na_2_CO_3_ | 60 | 70 |
| 7 | 0.25 | Water:EtOH/ 75 | Na_2_CO_3_ | 60 | 60 |
| 8 | 0.5 | EtOH/ 75 | Na_2_CO_3_ | 60 | 35 |
| 9 | 0.5 | Water/ 75 | Na_2_CO_3_ | 60 | 65 |
| 10 | 0.5 | DMF/ 75 | Na_2_CO_3_ | 60 | 45 |
| 11 | 0.5 | CH_3_CN/ 75 | Na_2_CO_3_ | 60 | 30 |
| 12 | 0.5 | THF/ 75 | Na_2_CO_3_ | 60 | 35 |
| 13 | 0.5 | Toluene/ 75 | Na_2_CO_3_ | 60 | Trace |
| 14 | 0.5 | Water:EtOH/ 75 | K_2_CO_3_ | 60 | 65 |
| 15 | 0.5 | Water:EtOH/ 75 | KOH | 60 | Trace |
| 16 | 0.5 | Water:EtOH/ 75 | NaOH | 60 | Trace |
| 17 | 0.5 | Water:EtOH/ 75 | Cs_2_CO_3_ | 60 | 80 |
| 18 | 0.5 | Water:EtOH/ 75 | None | 120 | Trace |
| ^a^ Reaction were run in 5.0 mL solvent with 1.0 mmol iodobenzene, 1.2 mmol boronic acid, and 2.0 mmol base.  ^b^ Isolated yield. | | | | | |

| **Table S2**  Optimization of conditions in the Sonogashira coupling reaction of bromobenzene **1** with phenyl acetylene **4^a^** | | | | | |
| --- | --- | --- | --- | --- | --- |
| **Entry** | **Loading of Catalyst**  **(mol%)** | **Conditions**  **Solvent/Temperature (°C)** | **Base** | **Time (h:min)** | **Yeild^b^ (%)** |
| 1 | 0.35 | Water/ 50 | Na_2_CO_3_ | 01:20 | 98 |
| 2 | 0.35 | EtOH/ 50 | Na_2_CO_3_ | 01:20 | 80 |
| 3 | 0.35 | CH_3_CN/ 50 | Na_2_CO_3_ | 01:20 | 30 |
| 4 | 0.35 | Toluene/ 50 | Na_2_CO_3_ | 01:20 | Trace |
| 5 | 0.35 | Water:EtOH/ 50 | Na_2_CO_3_ | 01:20 | 95 |
| 6 | 0.5 | Water/ 50 | Na_2_CO_3_ | 01:20 | 90 |
| 7 | 0.175 | Water/ 50 | Na_2_CO_3_ | 01:20 | 53 |
| 8 | 0.35 | Water/ 100 | Na_2_CO_3_ | 01:20 | 75 |
| 9 | 0.35 | Water/ r.t. | Na_2_CO_3_ | 01:20 | Trace |
| 10 | None | Water/ r.t. | Na_2_CO_3_ | 01:20 | Trace |
| 11 | 0.35 | Water/ 50 | K_2_CO_3_ | 01:20 | 80 |
| 12 | 0.35 | Water/ 50 | Cs_2_CO_3_ | 01:20 | 95 |
| 13 | 0.35 | Water/ 50 | KOH | 01:20 | 65 |
| 14 | 0.35 | Water/ 50 | NaOH | 01:20 | 25 |
| ^a^ Reaction were run with 1.0 mmol bromobenzene, 1.2 mmol phenyl acetylene, 2.0 mmol base, catalyst in 5.0 mL solvent.  ^b^ Isolated yields. | | | | | |

| **Table S3**  The study of loading and leaching of Pd element by ICP-AES analysis on the catalytic activity of the prepared catalysts in Sonogashira model reaction^a^ | | | | | |
| --- | --- | --- | --- | --- | --- |
| Entry | Catalyst | Loading Pd  (mmol.g^-1^) | Leaching Pd  (mmol.g^-1^) | Catalytic activity | |
|  |  |  |  | Time (h:min) | Yield^b^ (%) |
| 1 | Pd@h-Fe_2_O_3_ | 0.019 | 0.010 | 1:20 | 35 |
| 2 | Pd@h-Fe_2_O_3_@MRF | 0.030 | 0.019 | 1:20 | 70 |
| 3 | Pd@h-Fe_2_O_3_@MRF-C | 0.045 | 0.0079 | 1:20 | 88 |
| 4 | Pd@h-Fe_2_O_3_@glu | 0.023 | 0.020 | 1:20 | 50 |
| 5 | Pd@h-Fe_2_O_3_@glu-MRF | 0.039 | 0.013 | 1:20 | 80 |
| 6 | Pd@h-Fe_2_O_3_@glu-RF | 0.034 | 0.014 | 1:20 | 80 |
| 7 | Pd@h-Fe_2_O_3_@glu-RF-C | 0.066 | 0.0052 | 1:20 | 92 |
| 8 | Pd@h-Fe_2_O_3_@C | 0.075 | 0.0031 | 1:20 | 95 |
| ^a^ Reaction condition: aryl halides (1.0 mmol), terminal alkynes (1.2 mmol), catalyst (0.35 mol%), Na_2_CO_3_ (2.0 mmol) in H_2_O (5.0 mL) at 50 ˚C  ^b^ Isolated yields. | | | | | |

| **Table S4**  The comparison of the catalytic activity of Pd@h-Fe_2_O_3_@C with previously reported catalysts for the synthesis of **3a** (1-3)^a^ and **5a** (5-7)^b^. | | | | | | | |
| --- | --- | --- | --- | --- | --- | --- | --- |
| **Entry** | **Catalyst** | **Conditions** | **Solvent** | **base** | **Time**  **(h:min)** | **Yield**  **(%)^c^** | **Ref.** |
| 1 | CAGO catalyst | 80 °C | EtOH | K_2_CO_3_ | 08:00 | 94 | ^1^ |
| 2 | mTEG-CS-Co-Schiff-base | 90 °C | Water | K_2_CO_3_ | 01:00 | 93 | ^2^ |
| 3 | Pd(0)-TBA@biochar | 80 °C | PEG-400 | Na_2_CO_3_ | 02:05 | 96 | ^3^ |
| 4 | Pd@h-Fe_2_O_3_@C | 75 °C | Water:EtOH | Na_2_CO_3_ | 01:00 | 95 | This work |
| 5 | Pd^0^@CS/Al-Mt | 110 °C | DMSO/EG^d^ | CH_3_CO_2_K | 06:00 | 60 | ^4^ |
| 6 | Pt-MSHSs-Cu | 80 °C | THF | Et_3_N | 08:00 | 91/5 | ^5^ |
| 7 | Co@imine-POP | 80 °C | PEG | KOH | 10:00 | 72 | ^6^ |
| 8 | Pd@h-Fe_2_O_3_@C | 50 °C | Water | Na_2_CO_3_ | 01:20 | 98 | This work |
| ^a^ A mixture of iodobnzene, boronic acid, base and catalyst in solvent under thermal conditions.  ^b^ A mixture of bromobenzene, phenyl acetylene, base and catalyst in solvent under thermal conditions.  ^c^ Isolated yields.  ^d^ Ethylene glycol. | | | | | | | |

**References**

1 Anuma, S., Mishra, P. & Bhat, B. R. Copper complex with N-, O-architecture grafted graphene oxide nanosheet as a heterogeneous catalyst for Suzuki cross coupling reaction. *J Taiwan Inst Chem Eng* **95**, 643-651, doi:<https://doi.org/10.1016/j.jtice.2018.09.029> (2019).

2 Sobhani, S., Moghadam, H. H., Skibsted, J. & Sansano, J. M. A hydrophilic heterogeneous cobalt catalyst for fluoride-free Hiyama, Suzuki, Heck and Hirao cross-coupling reactions in water. *Green Chem.* **22**, 1353-1365, doi:<https://doi.org/10.1039/C9GC03455B> (2020).

3 Moradi, P., Hajjami, M. & Valizadeh-Kakhki, F. Biochar as heterogeneous support for immobilization of Pd as efficient and reusable biocatalyst in C–C coupling reactions. *Appl Organomet Chem* **33**, e5205, doi:<https://doi.org/10.1002/aoc.5205> (2019).

4 Zhao, J. *et al.* Chitosan supported Pd0 nanoparticles encaged in Al or Al-Fe pillared montmorillonite and their catalytic activities in Sonogashira coupling reactions. *Appl. Clay Sci.* **195**, 105721, doi:<https://doi.org/10.1016/j.clay.2020.105721> (2020).

5 Abolhosseini Shahrnoy, A. *et al.* Step‐by‐step synthesis of copper (I) complex supported on platinum nanoparticle‐decorated mesoporous silica hollow spheres and its remarkable catalytic performance in Sonogashira coupling reaction. *Appl. Organomet* **34**, e5645, doi:<https://doi.org/10.1002/aoc.5645> (2020).

6 Hajipour, A. R. & Khorsandi, Z. Pd/Cu‐free Heck and Sonogashira coupling reactions applying cobalt nanoparticles supported on multifunctional porous organic hybrid. *Appl Organomet Chem* **34**, e5398, doi:<https://doi.org/10.1002/aoc.5398> (2020).
